# Analysis of polychlorinated biphenyls (PCBs) in dairy products by modified QuEChERS/GC‐QqQ‐MS/MS method: A risk assessment study

**DOI:** 10.1002/fsn3.3269

**Published:** 2023-03-10

**Authors:** Amin Kiani, Majid Arabameri, Nabi Shariatifar, Abbas Mehraie, Fahimeh Tooryan, Ali Ghanbariasad, Saeed Shahsavari

**Affiliations:** ^1^ Department of Public Health, School of Public Health Fasa University of Medical Sciences Fasa Iran; ^2^ Food and Drug Laboratory Research Center Food and Drug Administration, Ministry of Health and Medical Education Tehran Islamic Republic of Iran Tehran Iran; ^3^ Department of Environmental Health Engineering, School of Public Health Tehran University of Medical Sciences Tehran Iran; ^4^ Department of Food Hygiene and Aquaculture, Faculty of Veterinary Medicine Ferdowsi University of Mashhad Mashhad Iran; ^5^ Department of Food Hygiene, Faculty of Veterinary Medicine Amol University of Special Modern Technologies Amol Iran; ^6^ Preventive Veterinary Medicine Graduate Group, School of Veterinary Medicine University of California Davis USA; ^7^ Department of Medical Biotechnologies Fasa University of Medical Sciences Fasa Iran; ^8^ Health Products Safety Research Center Qazvin University of Medical Sciences Qazvin Iran; ^9^ Department of Epidemiology and Biostatistics, School of Public Health Tehran University of Medical Sciences Tehran Iran

**Keywords:** doogh, kashk, Monte Carlo simulation (MCS), non‐dioxin‐like polychlorinated biphenyls (NDL‐PCBs), yogurt

## Abstract

Polychlorinated biphenyls (PCBs) are harmful chemicals that are persistent in the environment and can accumulate in the food chain. The purpose of the present research was to assess non‐dioxin‐like polychlorinated biphenyls (NDL‐PCBs) in some dairy products (yogurt, doogh, and kashk) using modified QuEChERS (Quick, Easy, Cheap, Effective, Rugged, and Safe) technique and gas chromatography–triple‐quadrupole mass spectrometry (GC‐QqQ‐MS/MS) method and risk assessment study. The LOQs (limit of quantifications), LODs (limit of detections), recovery, and RSD for the PCB analytes were 0.180–0.360, 0.06–0.12 ng/g fat, 97.45–102.63%, and 6.33–8.86%, respectively. The results revealed that the mean concentrations of Ʃ6‐NDL‐PCBs in samples were 15.17 ± 3.44 ng/g fat, which was lower than the standard level established by European Union (EU, 40 ng/g fat). The maximum mean level was PCB 180 (9.98 ± 2.04 ng/g fat) and the minimum mean level of PCBs in samples was PCB 28 (0.09 ± 0.06 ng/g fat). Also, results showed that kashk samples had a maximum mean level of 6‐NDL‐PCBs (18.66 ± 2.42 ng/g fat) and doogh samples had a minimum mean level of 6‐NDL‐PCBs (12.21 ± 2.22 ng/g fat). The mean level of 6‐NDL‐PCBs in yogurt samples was 14.65 ± 2.02 ng/g fat. The heat map results showed the correlation between the spectral indices of 6‐NDL‐PCBs in different dairy products. According to the Monte Carlo method, risk assessment was done using calculating the Estimated Daily Intake (EDI) and Incremental Life Cancer Risk (ILCR). The EDI values of 6 NDL‐PCBs based on the 95th percentile in yogurt, doogh, and kashk were 14.3, 1.49, and 0.5 ng/kg.day, respectively. Considering that the contaminant level in the samples is lower than the EU limit, it can be concluded that dietary exposure to 6 NDL‐PCBs may not pose a risk to the health of consumers.

## Graphical abstract

The purpose of the present research was to assess non‐dioxin‐like polychlorinated biphenyls (NDL‐PCBs) in some dairy products (yogurt, doogh, and kashk) using modified QuEChERS (Quick, Easy, Cheap, Effective, Rugged, and Safe) technique and gas chromatography–triple‐quadrupole mass spectrometry (GC‐QqQ‐MS/MS) method and risk assessment study. The LOQs (limit of quantifications), LODs (limit of detections), and recovery for the PCB analytes were 0.180–0.360, 0.06–0.12 ng/g fat, and 97.45–102.63%, respectively. The results revealed that the mean concentrations of Ʃ6‐NDL‐PCBs in samples were 15.17 ± 3.44 ng/g fat which was lower than the standard level established by European Union (EU, 40 ng/g fat). Also, results showed that kashk samples had a maximum mean level of 6‐NDL‐PCBs (18.66 ± 2.42 ng/g fat) and doogh samples had a minimum mean level of 6‐NDL‐PCBs (12.21 ± 2.22 ng/g fat). The mean level of 6‐NDL‐PCBs in yogurt samples was 14.65 ± 2.02 ng/g fat.

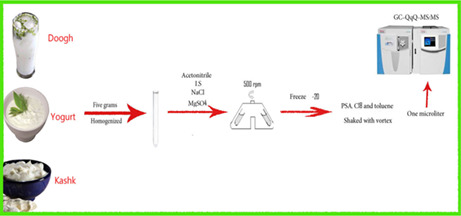

## 1 INTRODUCTION

People are constantly exposed to different types of anthropogenic pollutants in different ways. Today, food can be contaminated in different ways, and one of these ways is through the use of different pesticides (Ali & Aboul‐Enein, 2001, 2002; Ali et al., 2008; Al‐Shaalan et al., 2019; Jain & Ali, 1997). Some chemicals are able to be quickly excreted from the human body, while many of the fat‐soluble compounds are stored in different tissues of the body and are gradually metabolized and excreted (Kargarghomsheh et al., 2023; Naghashan et al., 2022). PCB is one of the fat‐soluble substances that remain in the body for a long time (Ahmadkhaniha et al., 2017; Ahmadloo et al., 2019; Yaminifar et al., 2021). Due to their stability and lipophilicity, PCBs preferentially accumulate in adipose tissue and therefore can be hazardous to human health (Lv et al., 2022; Pang et al., 2021). The main routes of exposure to dioxins and PCBs are the skin, inhalation, and gastrointestinal tract in humans, and 90% of human exposure is through the gastrointestinal tract. Swallowing and eating food is one of the most important routes of PCB absorption (Ahmadkhaniha et al., 2017; Cheney et al., 2019; Costabeber et al., 2018; Elangovan et al., 2019). PCBs are produced by chlorinating aromatics (12–68%), and some are commercially known as Aroclor, Phenoclor, and Clophen. There are 209 PCBs, among which six are non‐dioxin‐like PCBs (PCB‐180, ‐153, ‐138, ‐101, ‐52, and PCB‐28) have been selected as indicator PCBs (in‐PCBs) for residue monitoring in the environment (water, air, and soil) and in foods (such as dairy products, meat, etc.) (Amiridou & Voutsa, 2011; Bordajandi et al., 2004; Costabeber et al., 2018). Dioxin and PCB exposure has been related to immunological and neurobehavioral abnormalities in children, as well as diabetes and cancers. The IARC (International Agency for Research on Cancer) and WHO (World Health Organization) have classified DDT (Dichlorodiphenyltrichloroethane), HCB (hexachlorobenzene), and HCH (hexachlorocyclohexane) as group 2B or possible carcinogenic substances for humans and PCBs as 2A or probable carcinogenic substances ((IARC), I. A. f. R. o. C, 2017; (WHO), W. H. O, 1999; Ahmadkhaniha et al., 2017; Ahmadloo et al., 2019; Dedibegović et al., 2019).

Milk and dairy products are the key sources of human exposure to PCBs. PCBs enter the body of milk‐producing livestock mainly through the consumption of contaminated water and fodder (Özdemir et al., 2019; Roszko et al., 2014; Saktrakulkla et al., 2020; Schecter et al., 2010). Yogurt is a well‐known product obtained from the fermentation of milk. Two products made directly from yogurt include doogh and kashk (Kiani et al., 2018; Shiroodi et al., 2012). Doogh is a traditional dairy‐based beverage made from a combination of yogurt set or stirred, water, and salt. Despite the fact that doogh (yogurt‐based drink) is widely drunk around the world, particularly in Asia, there are clear distinctions between it and comparable products used in Europe owing to the variation in viscosity and the presence of salt (Kiani et al., 2018, 2019; Kouhpayeh et al., 2017). Humans have known for thousands of years that drying causes food to be stored for longer. For example, the main purpose of preparing yogurt in powder form (kashk) is easy and stable maintenance of the product. kashk is a concentrated boiled doogh or yogurt that contains a salt additive that comes in flat or round rolls and then dried in the presence of air (Iranmanesh et al., 2018; Shiroodi et al., 2012).

Today, many techniques are applied to detect PCBs in food products, such as gas chromatography–electron capture detector (GC‐ECD), gas chromatography–mass spectrometry (GC–MS), gas chromatography–triple‐quadrupole mass spectrometry (GC‐QqQ‐MS/MS), and gas chromatography coupled with high‐resolution mass spectrometry (GC–HRMS). Among them, GC‐QqQ‐MS/MS has high sensitivity and high accuracy and has many applications in this field (Reddy et al., 2019; Yaminifar et al., 2021). To evaluate PCBs and other contaminants in food products, a method was introduced in 2003 that was quick, easy, cheap, effective, rugged, and safe, called the QuEChERS technique (Mokhtari et al., 2002; Sereshti et al., 2022; Wu et al., 2022). The QuEChERS technique was used to extract analytes from the food matrix (Chen et al., 2022; Özdemir et al., 2019; Rutkowska et al., 2018).

Considering the lack of comprehensive research on the assessment of PCB levels in yogurt, kashk, and doogh of Iran, and the relatively high consumption of these products in the basket of Iranian, determining the concentration of 6 NDL‐PCBs in samples is necessary. For the first time in Iran and other countries, the current research was done to evaluate the concentration of 6 NDL‐PCBs in three dairy products (including doogh, kashk, and yogurt) by using modified QuEChERS (quick, easy, cheap, effective, rugged, and safe) extraction and GC‐QqQ‐MS/MS technique in different types of dairy products (yogurt, doogh, and kashk) and estimated the possible health risk posed by dairy products ingestion.

## 2 MATERIALS AND METHODS

### 2.1 Sample collection

A total number of 40 samples of yogurt, 40 samples of doogh, and 40 samples of kashk were analyzed. These samples have been obtained from the market of Tehran, Iran.

### 2.2 Chemicals and reagents

The PCB analytical standards with a purity degree more than 99% (PCB 180, 2,2′,3,4,4′,5,5′‐heptachlorobiphenyl; PCB 138, 2,2′,3,4,4′,5′‐hexachlorobiphenyl; PCB 153, 2,2′,4,4′,5,5′‐hexachlorobiphenyl; PCB 101, 2,2′,4,5,5′‐Pentachlorobiphenyl; PCB 52, 2,2′,5,5′‐tetrachlorobiphenyl and PCB 28, 2,4,4′‐trichlorobiphenyl) were obtained from Sigma‐Aldrich (USA). Methylbenzene, acetonitrile, methyl alcohol, MgSO_4_, toluene, primary secondary amine (PSA), and NaCl were obtained from Merck Co. (Germany).

### 2.3 Preparation of standard solution

Thirty‐two microliters of the solution of original standard (10 ng/μL of 6 PCBs with EC Number of 208‐759‐1) to prepare the standard stock solution was transferred with a micropipette to laboratory dishes and reached to 10 mL volume with toluene (Yaminifar et al., 2021).

### 2.4 Preparation of sample

Sample preparation of yogurt, doogh, and kashk was performed by using a QuEChERS modified method. First, 5 g of samples was homogenized and poured into a 50‐mL tube (glassy). Afterward, acetonitrile (20 mL) and 10 μL of internal standard (PCB29 with a concentration of 10 ng/mL) were added and mixed with vortex. Afterward, 4 mg of MgSO_4_ and 2 mg of NaCl were added to the tube (glassy) and shaken by vortex (vigorously). The solution was centrifuged at 5000 rpm for 10 min. Then, the above solution (10 mL) was poured into a 15‐mL vial (glassy). Next, the solution was placed in a freezer at −20°C for 15 min. Then, 400‐mg primary secondary amine (PSA), 40‐mg C18, and 1‐mL toluene were added to vial (glassy). Afterward, poured into a glass tube and shaken for 30 s with a vortex and standing for 5 min. Finally, 1 μL was injected into GC–MS/MS (Wang et al., 2016).

### 2.5 Analytical conditions and instrumentals

The 6 NDL‐PCB congeners were evaluated by GC–MS/MS device (Shimadzu GCMS‐TQ8040). For the separation of 6 NDL‐PCBs, the column Rxi‐5 MS, 30 meters × 0.25 millimeters × 0.25 micrometers was applied. The column oven temperature was 50 °C (1 min), 25°C per minute, 125°C−10°C per minute, and 300°C (3.5 min). The splitless was the mode of injection and the temperature of injection was 250°C. The argon was gas of CID and the mode of flow control was linear velocity (47.2 cm/sec). The voltage of the detector was tuning result +0.6 kv. The temperature of ion source and interface were 200°C and 250°C, respectively (Wang et al., 2016).

### 2.6 Quantification method

Quantification method (precision, recovery, linearity, accuracy, LOQ, and LOD) was done based on our past research (Yaminifar et al., 2021). To the linearity research, five samples (in series) were prepared by adding standard concentrations (0.10–40 ng/mL) on different days (in triplicate) and then injected into the GC–MS/MS in duplicate. Additionally, for each triplicate, two control negative samples were prepared, one without sample and the second without matrix. The acquired data were utilized to draw a calibration curve for each of the assessed PCB analytes. By the estimation of the RSD (relative standard deviation), the precision was assessed, also known as the CV (coefficient of variation), which was less than 15.94%, showing the effectiveness of the technique for repeatability (intra‐day) and for accuracy of intermediate (inter‐day). By the contamination with 8 ng/mL of each PCB analyte under analysis, five samples (equally) were ready for repeatability. The mentioned samples were assessed based on the suggested technique, from the extraction phase (all on the same day) until analysis by the GC–MS/MS. For the research accuracy, the standard addition method was utilized, involving adding different identified quantities of the certified standards of each analyte into the matrix (before the sample preparation). For each PCB analyte, four samples were ready by the addition of standard at concentrations of 20, 12, 8, and 0 ng/mL in triplicate and the determined quantities were associated with the amounts added. The LOD and LOQ were evaluated by the average blank value technique.

### 2.7 Assessment of human health risk

The present study examined the daily intake and carcinogenic risks owing to the Iranian dairy product consumption (yogurt, doogh, and kashk) contaminated with PCB. The model for computing risk was performed according to the previous report (Kiani, Arabameri, et al., 2021; Yaminifar et al., 2021). Briefly, the average daily intake is used to measure the oral exposure of harmful materials and assessed by the following equation (Kiani, Ahmadloo, et al., 2021; Kiani, Arabameri, et al., 2021):

(1) $$\mathrm{EDI}=\frac{C\times \mathrm{ED}\times \mathrm{EF}\times \mathrm{IR}}{\mathrm{BW}\times \mathrm{AT}}$$

In this equation, estimated daily intake (EDI) is based on the ng/kg.day (Eghbaljoo‐Gharehgheshlaghi et al., 2020), C is the PCB concentration based on ng/g fat, the definition and description of the above variables are shown in Table 1. To evaluate the cancer risk of exposure of PCB compounds, the incremental lifetime carcinogenic risk (ILCR) model was obtained by the following equation (Kiani, Ahmadloo, et al., 2021).

(2) ILCR=EDI×SF

**TABLE 1:** Parameters applied in the present study for health exposure assessment in dairy products.

Exposure parameters		Unit	Reference
SF	Carcinogenic slope factor of oral intake (2)	(mg (kg/d))^−1^	[45]
C	concentrations of PCBs	μg/kg	–
EDI	estimated daily intake	mg/kg	[46]
EFi	exposure frequency	days per year	[47]
IR	Average daily intake	kg/day	[48]
ED	exposure duration	days	[49]
AT	average time	days	[6]
BW	body weight (for children and adults is between 15 and 70)	kg	[50]

By the guidance of US EPA, where SF is equal to 2 mg/Kg b.w. per day for PCBs was referenced (Falandysz et al., 1999). The definition and description of variables are shown in Table 1.

### 2.8 Statistical analysis

By using SPSS (ver. 25), a practical and easy statistical analysis was utilized for the values of standard deviation (SD), mean, maximum, and minimum. The test of Kolmogorov–Smirnov was used to evaluate the normal distribution of continuous variables. ANOVA, independent t‐test, Mann–Whitney, and Kruskal–Wallis were utilized to evaluate differences between groups. A p‐value less than 0.05 was selected as the significant difference for all tests. A data visualization procedure was conducted to explain the correlation among the variables and highlight dairy products' contamination (Arabameri et al., 2019; Moradi et al., 2021). The heat map was performed in evaluating the relationships of contamination among samples online at the site https://biit.cs.ut.ee/clustvis/.

## 3 RESULTS AND DISCUSSION

### 3.1 Analytical method evaluation performance

Analytical characteristics of applied techniques for the examination of PCB compounds are shown in Table 2. The results revealed that the LOQs and LODs for the PCB analytes were 0.180–0.360 and 0.06–0.12 ng/g fat, respectively. Besides, the percent of RSD and recovery of the 6 NDL‐PCB analyte were found at a range of 6.33–8.86 and 97.45–102.63%, respectively. Our findings are almost similar to the research of Yaminifar et al. who studied the PCB compounds in butter samples with QuEChERS modified method and GC–MS device, the recovery obtained in their results was 91.2–107.14% (Yaminifar et al., 2021). This similarity could be due to the use of a similar method in measuring PCBs.

**TABLE 2:** The linear range of concentration, LOQ (limit of quantifications), LOD (limit of detection), recovery, RSD (relative standard deviation), and inter‐day and intra‐day reproducibility of validation method.

Compound	Linear range of concentration (ng/mL)	LOD (ng/g)	LOQ (ng/g)	RSD%	Recovery%	Intra‐day	Inter‐day
PCB 28	0.10–40	0.080	0.250	6.33	100.21	7.55	10.58
PCB 52	0.10–40	0.080	0.250	8.86	102.63	10.34	14.92
PCB 101	0.10–40	0.120	0.360	7.54	98.49	11.44	13.81
PCB 138	0.10–40	0.060	0.180	7.49	97.45	9.58	14.98
PCB 153	0.10–40	0.100	0.310	8.55	101.38	10.81	15.22
PCB 180	0.10–40	0.080	0.250	8.29	98.97	11.83	15.94

### 3.2 Evaluation of PCBs in dairy product samples

Table 3 shows the statistical analysis of our results, which shows the mean, maximum, and minimum of each PCB analyte in yogurt, doogh, and kashk samples. The outcomes of our research exhibited that the average level of Ʃ6‐NDL‐PCBs in samples was 15.17 ± 3.44 ng/g fat, which was lower than the standard levels established by EU (40 ng/g fat). The maximum mean level was PCB180 (9.98 ± 2.04 ng/g fat) and the minimum mean level of PCBs in samples was PCB28 (0.09 ± 0.06 ng/g fat).

**TABLE 3:** Statistical analysis of PCBs in dairy product samples (ng/g fat).

	Minimum	Maximum	Mean	Std. deviation (SD)
PCB 28	0.04	0.26	0.09	0.06
PCB 52	0.04	0.9	0.19	0.21
PCB 101	0.06	0.18	0.1	0.05
PCB 138	0.24	0.76	0.48	0.15
PCB 153	2.65	6.75	4.33	1.23
PCB 180	6.28	13.42	9.98	2.04
Total	9.62	21.71	15.17	3.44

It should be noted that so far no studies have been done on the amount of these contaminants (PCBs) in doogh and kashk, only a few studies have been done on yogurt and other dairy products. Table 4 compares the levels of NDL‐PCB analytes in milk and dairy products in other researches (all over the world) with the current research. This table demonstrates exception of four articles, which had upper results, other articles had less outcomes than this article. This table also showed that the lower the thinning of a matrix (more water than other products), the lower its fat content and the fewer processes performed on it, that product will have less amount of pollutants (like milk). Also, in comparison with other investigators, the results of PCB contamination in the present study showed that environmental pollutant (water, soil, and air) with pesticides, petroleum products, contaminants of diesel trucks and paints may cause contamination of livestock feed and products of agriculture like mentioned dairy products. Additionally, the close proximity of farms of animals to the oil refineries, roads, and industries may be the reason for livestock pollution, followed by livestock products like these dairy products. Generally, these compound levels (6 NDL‐PCBs) in the samples of yogurt, doogh, and kashk in Tehran city that was lower than the standard levels established by EU (Ahmadloo et al., 2019; Hoogenboom et al., 2020; Malisch & Dilara, 2007; Nardelli et al., 2020; Saktrakulkla et al., 2020; Yaminifar et al., 2021).

**TABLE 4:** Comparison of the current research with other studies.

Researcher	Year	Country	Compounds	Samples	Results	Compared with our study	References
Shahsavari et al.	2022	Iran	NDL‐PCBs	Cream and ice cream samples	21.634 ± 2.18 and 12.317 ± 1.524 ng/g fat, respectively	In cream higher than and in ice cream lower than our results	[51]
Son et al.	2012	South Korea	NDL‐PCBs	Milk, ice cream, yogurt, cheese, and butter	0.09, 0.189, 0.133, 0.161, and 0.449 ng/g fat, respectively	Lower than present results	[41]
Schecter et al.	2010	USA	NDL‐PCBs	Frozen yogurt, cheese and cream cheese, whole milk, butter, and ice cream	nondetected (ND) in all samples	Lower than present results	[18]
Witczak A et al.	2019	Canada and Denmark	NDL‐PCBs	Milk and yogurt	1.858 and 1.754 ng/g fat, respectively	Lower than present results	[42]
Yaminifar et al.	2021	Iran	NDL‐PCBs	Butter samples	21.701 ± 9.02 ng/g fat	Higher than present results	[3]
Uçar et al.	2011	Turkey	NDL‐PCBs	Butter samples	0.20–3.04 ng/g fat	Lower than present results	[52]
Cimenci et al.	2013	Belgium	NDL‐PCBs	Butter and milk samples	4.72 ng/g fat for both of them	Lower than present results	[53]
Santos J. et al.	2006	Brazil	NDL‐PCBs	Industrialized and homemade cheese samples	33.32 ± 25.40 ng/g fat and 26.58 ± 9.91 ng/g fat, respectively	Higher than present results	[54]
Pérez et al.	2012	Mexico	NDL‐PCBs	Milk samples	9.6 ng/g fat	Lower than present results	[55]
Atmaca	2019	Turkey	NDL‐PCBs	Milk samples	127.27 ng/g fat	Higher than present results	[56]
Miclean M et al.	2018	Romania	NDL‐PCBs	Milk samples	9.12 ng/g fat	Lower than present results	[57]
Rusin et al.	2019	Poland	NDL‐PCBs	Milk samples	1.25 ng/g fat	Lower than present results	[44]

### 3.3 Evaluation of PCBs in different types of dairy products (yogurt, doogh, and kashk)

The results presented in Table 5 show the level of 6 NDL‐PCBs in different types of dairy product samples. Our results showed that kashk samples had a maximum mean level of 6‐NDL‐PCBs (18.66 ± 2.42 ng/g fat) and doogh samples had a minimum mean level of 6‐NDL‐PCBs (12.21 ± 2.22 ng/g fat). The mean level of 6‐NDL‐PCBs in yogurt samples was 14.65 ± 2.02 ng/g fat. So, in all samples, the level of 6‐NDL‐PCBs was lower than the EU standard level (40 ng/g fat). Since doogh is obtained by thinning yogurt and kashk from thickening yogurt, the reason for the lower results of doogh and the higher results of kashk can probably be explained. Therefore, it can be stated that with the concentration of dairy products, the amount of PCB compound should probably increase as well, although the amount of this increase will not be dangerous for the consumer because it is less than the existing standards. Son et al. (2012), Schecter et al. (2010) and Witczak and Mituniewicz‐Małek (2019) evaluated PCB concentrations in yogurt samples and reported that the mean level of Ʃ6‐NDL‐PCBs was 0.133, ND and 1.754 ng/g fat, respectively, that were less than this research (Schecter et al., 2010; Son et al., 2012; Witczak & Mituniewicz‐Małek, 2019).

**TABLE 5:** Statistical analysis of PCBs in different types of dairy products (ng/g fat).

Type	Yogurt				Doogh				Kashk				*p* value
	Min	Max	Mean	SD	Min	Max	Mean	SD	Min	Max	Mean	SD	
PCB 28	0.04	0.15	0.09	0.05	0.04	0.11	0.06	0.03	0.04	0.26	0.13	0.08	.82
PCB 52	0.04	0.24	0.14	0.07	0.04	0.9	0.25	0.37	0.09	0.34	0.17	0.1	1
PCB 101	0.06	0.13	0.09	0.04	0.06	0.14	0.08	0.04	0.06	0.18	0.12	0.06	.33
PCB 138	0.41	0.61	0.5	0.08	0.24	0.45	0.34	0.09	0.41	0.76	0.61	0.14	.33
PCB 153	3.24	5.88	4.26	0.99	2.65	4.07	3.33	0.62	4.21	6.75	5.41	1.09	.33
PCB 180	8.64	10.88	9.58	0.93	6.28	9.74	8.14	1.36	10.82	13.42	12.21	1.1	.08
Total	12.59	17.89	14.65	2.02	9.62	15.09	12.21	2.22	15.87	21.71	18.66	2.42	.08

### 3.4 Evaluation of PCBs in different brands

The outcomes revealed in Table 6 show the level of 6 NDL‐PCBs in different brands of dairy product samples. The results showed that brand A had the maximum mean level of 6 NDL‐PCBs (18.23 ± 3.32 ng/g fat) and brand E had the minimum mean level of 6 NDL‐PCBs (12.93 ± 2.78 ng/g fat). The reason for the higher level of PCBs in brand E can be the use of more polluted livestock fodder, the supply of milk from contaminated environments, the proximity of the factory to urban and industrial centers, the proximity of livestock to contaminated environments, and the use of contaminated containers and equipment.

**TABLE 6:** Statistical analysis of PCBs in different brands of dairy products (ng/g fat).

Brand	A			B			C			D			E			*p*‐value
	Min	Max	Mean ± SD	Min	Max	Mean ± SD	Min	Max	Mean ± SD	Min	Max	Mean ± SD	Min	Max	Mean ± SD	
PCB 28	0.11	0.26	0.17 ± 0.08	0.04	0.04	0.04 ± 0.00	0.04	0.09	0.07 ± 0.03	0.09	0.12	0.11 ± 0.02	0.04	0.15	0.08 ± 0.06	.52
PCB 52	0.24	0.9	0.49 ± 0.36	0.12	0.17	0.15 ± 0.03	0.04	0.09	0.06 ± 0.03	0.13	0.16	0.14 ± 0.02	0.04	0.11	0.09 ± 0.04	.1
PCB 101	0.13	0.18	0.15 ± 0.03	0.06	0.14	0.11 ± 0.04	0.06	0.06	0.06 ± 0.00	0.06	0.06	0.06 ± 0.00	0.06	0.17	0.1 ± 0.06	.52
PCB 138	0.45	0.76	0.61 ± 0.16	0.34	0.64	0.49 ± 0.15	0.41	0.7	0.55 ± 0.15	0.27	0.52	0.42 ± 0.13	0.24	0.41	0.35 ± 0.1	.1
PCB 153	3.75	6.75	5.46 ± 1.54	3.44	5.66	4.48 ± 1.12	4.05	6.06	4.73 ± 1.15	2.76	4.39	3.64 ± 0.82	2.65	4.21	3.37 ± 0.79	.52
PCB 180	9.74	13.42	11.35 ± 1.88	8.83	12.51	10.36 ± 1.92	8.54	12.97	10.05 ± 2.53	6.28	11.35	9.18 ± 2.61	7.31	10.82	8.95 ± 1.77	.52
Total	15.09	21.71	18.23 ± 3.32	12.83	19.16	15.63 ± 3.23	13.16	19.97	15.52 ± 3.86	9.62	16.58	13.55 ± 3.56	10.34	15.87	12.93 ± 2.78	.52

### 3.5 Human health risk assessment

The results presented in Table 7 show the daily intake of 6 NDL‐PCBs in dairy product samples. The yogurt had the maximum mean daily intake of 6 NDL‐PCBs (1.43 ng/kg day) and kashk had the minimum mean daily intake of 6 NDL‐PCBs (5.09E−1 ng/kg day). The reason for the higher consumption of yogurt in Tehran (Iran) was associated with an increased risk of NDL‐PCBs daily intake. The rank order of 6 NDL‐PCBs EDI values based on percentile 95% in yogurt was PCB 180 (2.99E−5) > PCB 153 (1.35E−5) > PCB 138 (1.59E−6) > PCB 52 (4.42E−7) > PCB 28 (2.82E−7) > PCB 101(2.79E−7); in doogh was PCB 180 (I2.54E−6) > PCB 153(1.08E−6) > PCB 138 (1.28E−7) > PCB 52 (3.68E−8) > PCB 101 (2.43E−8) > PCB 28 (2.34E−8); in kashk was PCB 180 (1.11E−6) > PCB 153 (4.80E−7) > PCB 138 (5.25E−8) > PCB 52 (1.51E−8) > PCB 28 (1.10E−8) > PCB 101(1.10E−8).

**TABLE 7:** Simulation results for EDI (estimated daily intake) of NDL‐PCBs (non‐dioxin‐like‐polychlorinated biphenyls) detected in dairy products (yogurt, doogh, and kashk).

	Percentiles	PCB 28	PCB 52	PCB 101	PCB 138	PCB 153	PCB 180	TOTAL NDL‐PCB
Yogurt	5%	5.96E−2	9.15E−2	6.01E−2	3.30E−1	2.78E+0	6.18E+0	9.72E+0
	50%	8.91E−2	1.37E−1	8.74E−2	4.95E−1	4.16E+0	9.26E+0	1.43E+1
	75%	1.06E−1	1.62E−1	1.03E−1	5.79E−1	4.87E+0	1.09E+1	1.68E+1
	95%	1.32E−1	2.09E−1	1.27E−1	7.28E−1	6.19E+0	1.45E+1	2.12E+1
Doogh	5%	3.32E−3	1.38E−2	4.31E−3	1.88E−2	1.78E−1	4.43E−1	6.54E−1
	50%	4.89E−3	2.06E−2	6.42E−3	2.76E−2	2.68E−1	6.68E−1	1.01E+0
	75%	5.79E−3	2.40E−2	7.63E−3	3.32E−2	3.17E−1	7.79E−1	1.19E+0
	95%	7.40E−3	3.08E−2	9.57E−3	4.11E−2	4.05E−1	9.65E−1	1.49E+0
Kashk	5%	2.36E−3	3.00E−3	2.16E−3	1.11E−2	9.60E−2	2.21E−1	3.40E−1
	50%	3.52E−3	4.54E−3	3.25E−3	1.70E−2	1.47E−1	3.34E−1	5.09E−1
	75%	4.19E−3	5.35E−3	3.85E−3	1.99E−2	1.72E−1	3.94E−1	6.05E−1
	95%	5.33E−3	6.88E−3	4.83E−3	2.57E−2	2.20E−1	5.02E−1	7.66E−1

**FIGURE 1:**
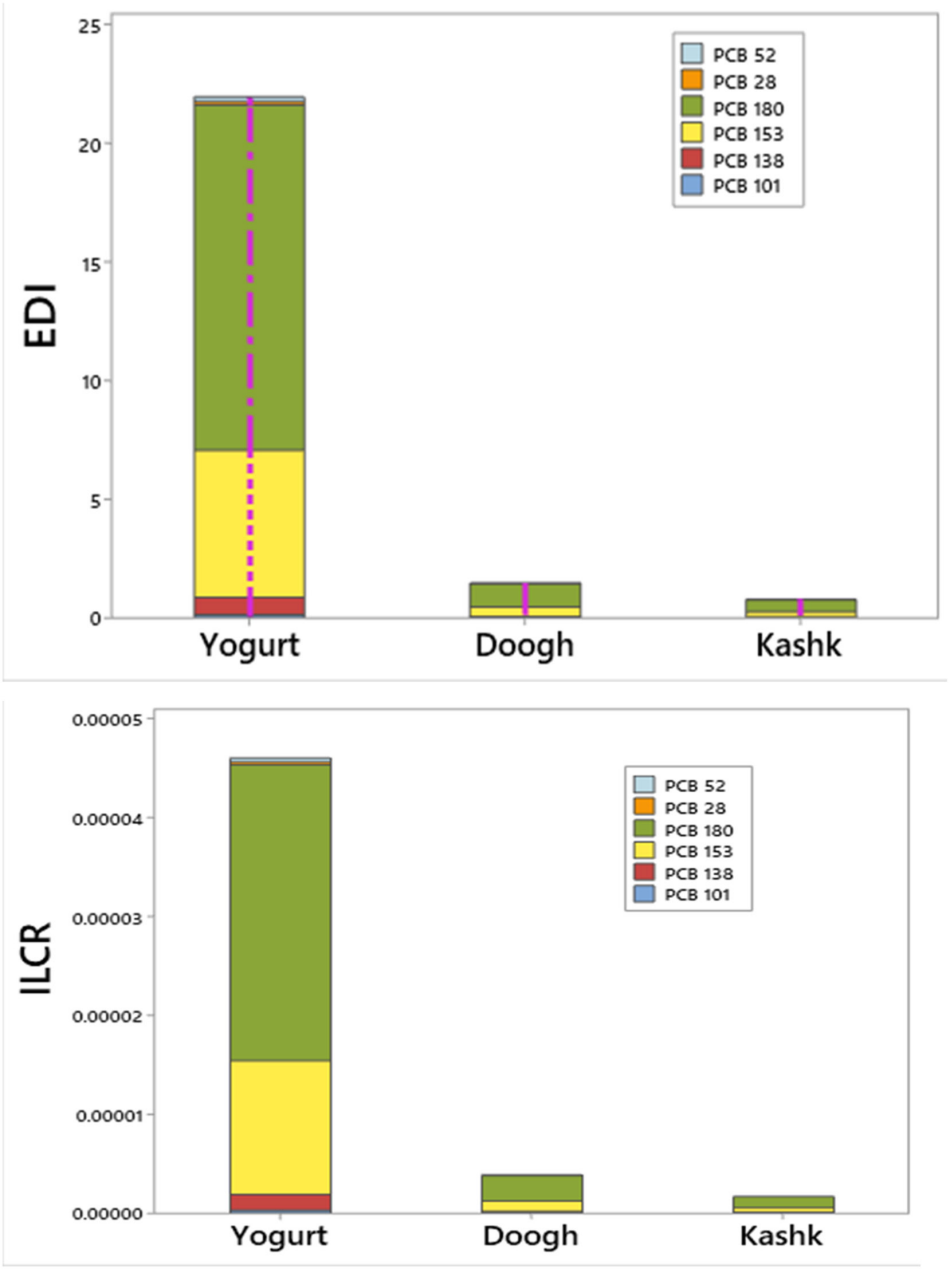
Contribution to overall Estimated Daily Intake (EDI) and Incremental Life Cancer Risk (ILCR) in dairy products (yogurt, doogh, and kashk).

The average dietary exposure to NDL‐PCBs was far below the WHO's tolerable daily intake (10 ng/kg BW/day) for the general population (Dinc et al., 2019). The EDI distribution among PCB congeners with the most and least contribution is shown in Figure 1. In all samples, PCB180 became the most important congener.

**FIGURE 2:**
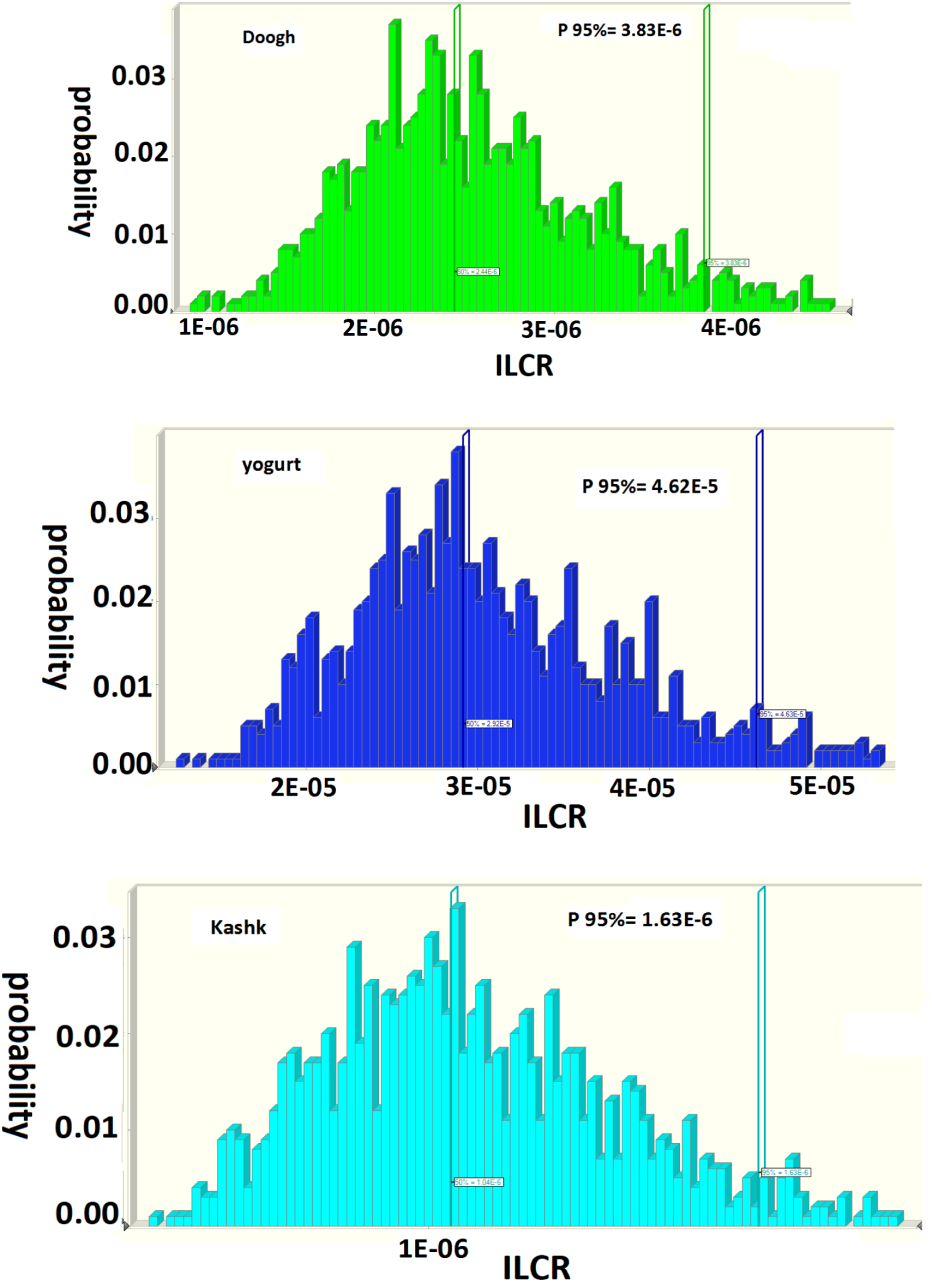
Simulation results for Incremental Life Cancer Risk (ILCR) of NDL‐PCB (non‐dioxin‐like‐polychlorinated biphenyls) detected in dairy products (yogurt, doogh, and kashk).

Previous studies assessed 6 NDL‐PCB levels in cow milk from Silesia Province, Poland and exposed that the mean level of 6 NDL‐PCBs was 2.1 ng/kg per day (Rusin et al., 2019). In China, Kang et al. noted that dietary exposure to PCBs in food was 26.47 ng/kg per day than our investigation (Kang et al., 2020). Also, Chung et al. reported that, in Hong Kong, the lower and higher bound for the intake of PCBs was 0.68 and 1.38 ng/kg per day, respectively (Chung et al., 2018). The people studied in Poland were moreover more exposed to NDL‐PCB compounds than in the current research. These variations are mostly due to the different levels of environmental pollution with persistent organic pollutants and different sources of these compound emissions. Other key diet components were not included in the estimates since they were based only on the use of dairy products. As a result, the total health risk from dietary exposure to a complete diet is likely to be upper. The results presented in Figure 2 show the amount of ILCR in different dairy product samples. The ILCR values from the Monte Carlo simulation showed that yogurt samples had a maximum mean level of ILCR (4.63E−5) and doogh samples had a minimum mean level of ILCR (1.63E−6) which shows their potency be a low risk of oral cancer for people in Iran. The cancer risk of PCBs for the target population was much lower than the unacceptable risk (10^−4^). The oral cancer risk of 6 NDL‐PCBs was lower than the unacceptable limit (10^−4^) for people in Iran. Rusin et al. measured a total of 18 PCBs (that consist of 6 NDL‐PCBs) in popular foods of animal origin that the mean cancer risk resulting from eggs, chicken meat, and farm‐fresh cow milk intake 6.9 × 10^−3^, 3.4 × 10^−4^, and 2.8 × 10^−4^, respectively, was higher than this research (Rusin et al., 2019).

### 3.6 Analysis based on heat map results

**FIGURE 3:**
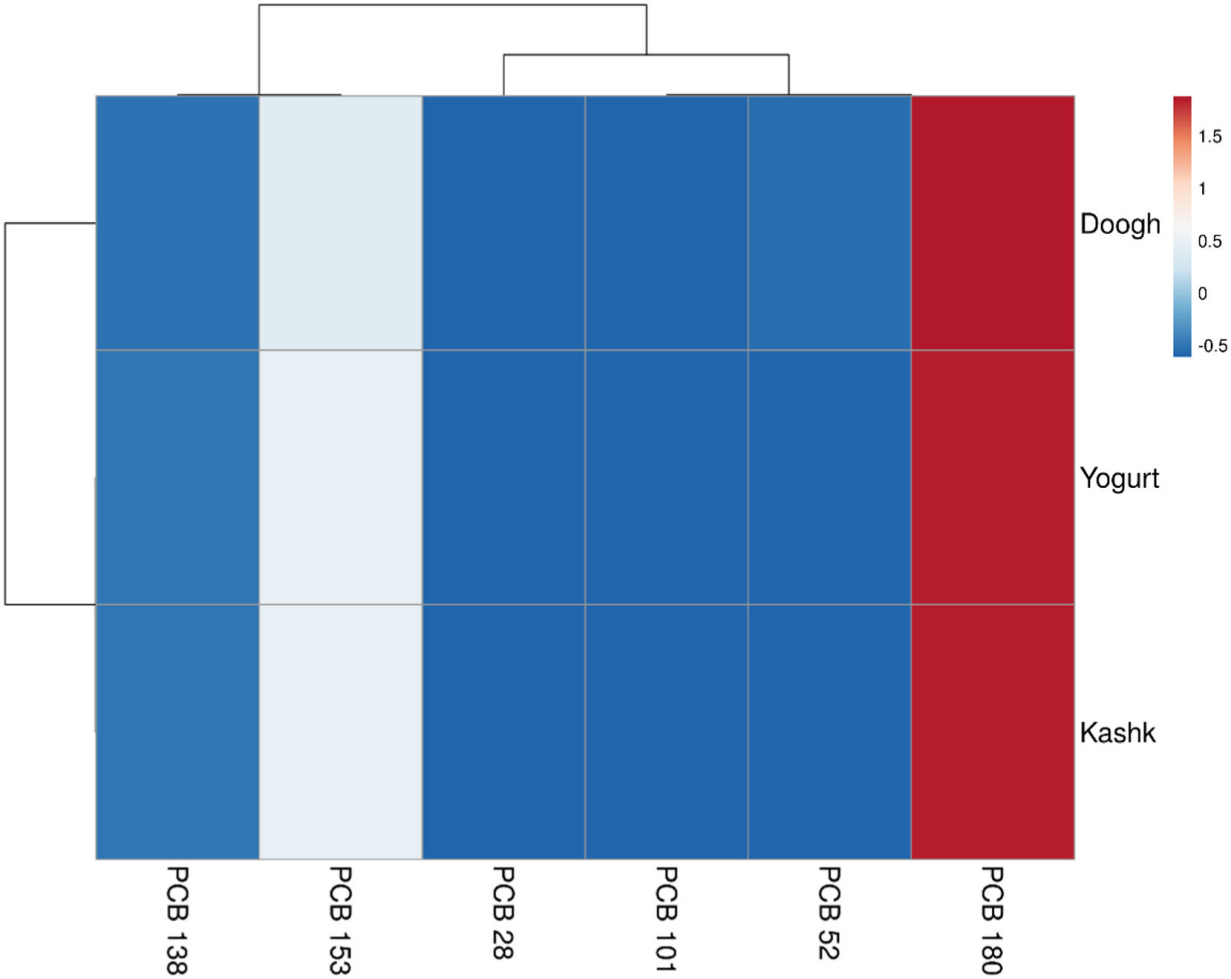
Heat map of 6 NDL‐PCBs (non‐dioxin‐like‐polychlorinated biphenyls) in dairy products (yogurt, doogh, and kashk).

The heat map provides a profile overview of the highermost and lowermost data in the matrix by clustering similar parameters. The dairy product contamination to PCB congeners was visually stated as a heat map for each sample. The patterns of the heat map were markedly varied depending on the PCB congener. The first cluster includes PCB 138 and PCB 153, and the second cluster contains two subgroups. The cluster dendrogram is shown in Figure 3. As the Euclidean space reductions, the samples showed a higher correlation. Hence, PCB 180, PCB 131, and PCB 52 had a high correlation among different samples.

## 4 CONCLUSION

Conclusively, this study was the first to compare 6 NDL‐PCB residues in different types of dairy product (yogurt, doogh, and kashk) samples of Tehran. The samples were analyzed using modified QuEChERS and by GC‐QqQ‐MS/MS method which had a very good performance in this regard with the recovery of more than 97.45%. Our study showed that PCB contaminant levels were higher in kashk samples and lower in doogh samples (kashk>yogurt>doogh). The 6 NDL‐PCB levels in all samples were lower than the EU standard level. The dietary exposure to NDL‐PCBs detected in dairy products was much lower than the recommended TDI (10 ng/kg BW/day). The Monte Carlo simulation output showed the index of ILCR owing to ingestion of 6‐NDL‐PCBs lower than the safe level (ILCR >1E−4). The limitation of the present study was that we analyzed only three dairy products, so it is suggested to evaluate and measure other dairy products in the future. Also, it is suggested that more control and monitoring be done on dairy products due to the high consumption and high sensitivity of these products.

## CONFLICT OF INTEREST STATEMENT

The authors of this article state that there is no competitive interest.

## ETHICAL APPROVAL

This article does not include any studies with human or animal participants conducted by any of the authors.

## Data Availability

The datasets generated during and/or analyzed during the current study are available from the corresponding author on reasonable request.
